# Combining biomechanical stimulation and chronobiology: a novel approach for augmented chondrogenesis?

**DOI:** 10.3389/fbioe.2023.1232465

**Published:** 2023-06-30

**Authors:** Judit Vágó, Roland Takács, Patrik Kovács, Tibor Hajdú, Daan R. van der Veen, Csaba Matta

**Affiliations:** ^1^ Department of Anatomy, Faculty of Medicine, Histology and Embryology, University of Debrecen, Debrecen, Hungary; ^2^ Chronobiology Section, Faculty of Health and Medical Sciences, University of Surrey, Guildford, United Kingdom

**Keywords:** chondrogenesis, circadian clock, chondrochronology, cartilage regeneration, biomechanical stimuli, osteoarthritis, chondrotherapy

## Abstract

The unique structure and composition of articular cartilage is critical for its physiological function. However, this architecture may get disrupted by degeneration or trauma. Due to the low intrinsic regeneration properties of the tissue, the healing response is generally poor. Low-grade inflammation in patients with osteoarthritis advances cartilage degradation, resulting in pain, immobility, and reduced quality of life. Generating neocartilage using advanced tissue engineering approaches may address these limitations. The biocompatible microenvironment that is suitable for cartilage regeneration may not only rely on cells and scaffolds, but also on the spatial and temporal features of biomechanics. Cell-autonomous biological clocks that generate circadian rhythms in chondrocytes are generally accepted to be indispensable for normal cartilage homeostasis. While the molecular details of the circadian clockwork are increasingly well understood at the cellular level, the mechanisms that enable clock entrainment by biomechanical signals, which are highly relevant in cartilage, are still largely unknown. This narrative review outlines the role of the biomechanical microenvironment to advance cartilage tissue engineering via entraining the molecular circadian clockwork, and highlights how application of this concept may enhance the development and successful translation of biomechanically relevant tissue engineering interventions.

## 1 Introduction

The Global Burden of Disease 2019 study has shown that over 1.5 billion people live with musculoskeletal conditions, including osteoarthritis (OA), rheumatoid arthritis (RA), low back pain, neck pain, fractures, and other injuries (Cieza et al., 2021). The global burden of OA poses a considerable impact on individuals, communities, and healthcare systems, and these are projected to increase further in the coming decades (Foster et al., 2023). There is no curative treatment available for patients with OA. Only moderate benefits have been observed following hyaluronan, glucocorticoid and platelet rich plasma intra-articular therapies for pain and function in knee OA (Rodriguez-Garcia et al., 2021). Exercise therapy has been identified as the best treatment for OA pain, followed by nonsteroidal anti-inflammatory drugs (NSAIDs) and opioids (Thorlund et al., 2022). Other effective therapies include the monoclonal antibody tanezumab, the antidepressant duloxetine, autologous microfragmented adipose tissue, intra-articular ketorolac injection, and subchondral or intra-articular mesenchymal stem cell (MSC) injection (Foster et al., 2023). MSCs are ideal candidates to repair damaged issues due to their trilineage differentiation potential, trophic effects, and immunomodulatory properties (Wei and Bao, 2022).

Pain in OA mainly occurs during the day and during physical activities, but patients may also experience resting pain at night (Fu et al., 2018), indicating that pain in OA may have a diurnal pattern. Pain patterns are different in RA compared to OA: RA patients usually exhibit a peak onset of pain in the morning, whereas the pain from OA worsens during the day (Knezevic et al., 2023). Such rhythmicity in pain may have important implications for patients, both in terms of planning their daily activities, and in developing more efficient chronotherapeutic programs (Bellamy et al., 2002). The circadian clock could also be exploited to increase the efficacy of MSC-based chondro-regenerative approaches (Vago et al., 2022). Although research in this area has shed some light on clock-controlled pathways in chondrocytes, we are far from using chronotherapy in OA patients with clinically relevant outcomes.

In this narrative review, we highlight current challenges in chondro-regenerative applications, and demonstrate that the biomechanical microenvironment could be exploited to fine-tune existing approaches via the modulation of the circadian clock.

## 2 Cartilage tissue engineering

Almost three decades ago, the emerging field of tissue engineering held the prospect of repairing injured tissues or organs (Huey et al., 2012). The original premise was that tissues with comparable qualities to those in the human body could be generated *in vitro* and implanted to the site of damage to restore function (Langer and Vacanti, 1993). Cartilage appeared as an ideal candidate, as it is avascular and is characterized by only a few cell types (Huey et al., 2012). However, regenerative tissue engineering has been more successfully applied in other tissues such as bone (Elgali et al., 2017). This is at least partially attributable to the recent understanding that there is a significant level of heterogeneity among chondrocyte populations (Wang et al., 2021).

Cartilage tissue engineering mostly relies on a combination of scaffolds (Roffi et al., 2017; Uzieliene et al., 2021) or hydrogels (Naranjo-Alcazar et al., 2023; Uzieliene et al., 2023), cells (Huang et al., 2016), and stimulatory factors (Kwon et al., 2016) including mechanical stimulation (Juhasz et al., 2014; Ouyang et al., 2019), as well as autologous or allogeneic cells. Universal donor cells that are invisible to the immune system are also on the horizon (Lanza et al., 2019). The properties of the scaffold, including structure, surface characteristics, and mechanical properties, are also important (Cengiz et al., 2018). The regenerative attributes of cells depend on *ex vivo* culturing parameters and external factors such as mechanical stimulation (Ouyang et al., 2019). Endogenous stem cells in an appropriate scaffold secrete bioactive molecules that provide a suitable microenvironment for controlling regeneration (Caplan, 2007).

Generally, *ex vivo* cultured cells are seeded onto scaffolds, and a bioreactor is used before implantation (Cengiz et al., 2018). However, seeding cultured cells might not even be necessary. Novel approaches are being developed to bypass the complicated *ex vivo* process. The patient’s own regenerating capacity can be exploited by mobilizing endogenous stem cells or tissue-specific progenitor cells. Implanted scaffolds may provide a suitable microenvironment to aid the recruitment of host cells that can in turn regenerate functional hyaline cartilage (Ko et al., 2013).

Stem cells are not the exclusive cell source for regenerative medicine. Most tissue engineering approaches rely on the assumption that stem cells contribute as building blocks to tissue regeneration (Altamirano et al., 2020). However, stem cells are being increasingly recognized to coordinate healing via their immunomodulatory capacity (Altamirano et al., 2020). Adipose or bone marrow-derived stem cells, or cells isolated from the target tissue are commonly used sources (Cengiz et al., 2018). Cell–scaffold interactions pose some of the questions that need to be resolved in order to translate these constructs from bench to bedside (Huey et al., 2012).

Bioreactors are also extensively applied to stimulate regenerative cell function (Ravichandran et al., 2018). These tools have an outstanding potential to grow and mature 3D tissues by providing conditions that mimic their native microenvironment. Development in this direction has a significant potential for clinical translation (Ravichandran et al., 2018).

## 3 The biomechanical microenvironment of developing and mature cartilage

The articular cartilage matrix exhibits a unique architecture which is challenging to regenerate *in vitro*. Each chondrocyte is surrounded by the pericellular matrix (PCM). The PCM is spatially distinct within the extracellular matrix (ECM) and serves as the biomechanical microenvironment (BME) of chondrocytes (Xu et al., 2022). The molecular composition of the PCM differs from the rest of the ECM, and confers diverse biomechanical properties to transform physical stimuli to molecular pathways (Guilak et al., 2006). Mechanical stimuli are vital in chondrocyte differentiation and joint formation, and also in mature articular cartilage (Jortikka et al., 1997), which highlights the importance of the BME at early stages of development (Vining and Mooney, 2017).

Different kinds of forces, including compression, shear stress and tensile strain were studied on articular chondrocytes (Grad et al., 2011; Khoshgoftar et al., 2018). Pressure applied to joint surfaces generates interstitial fluid flow that dynamically alters the amount of water and ions in the PCM/ECM, and this puts additional physical factors under the spotlight, such as shear stress caused by fluid flow, changes in local pH, osmotic and hydrostatic pressure (Hing et al., 2002; Elder and Athanasiou, 2009). Joint loading is a complex process *in vivo*, which brings challenges to mechanobiology research in terms of modelling the complexity of physical stimuli in developing, mature and pathological articular cartilage (O'Conor et al., 2013).

The biochemical composition of the BME makes chondrocytes sensitive to physical stimuli. Type VI collagen is essential in the PCM, and it acts as the main biomechanical transducer by anchoring chondrocytes to the matrix via integrin receptors (Alexopoulos et al., 2009). Type IV and IX collagens are also present in the PCM, in addition to special ground substance components such as proteoglycans and multi-adhesive glycoproteins (Schminke et al., 2016; Chu et al., 2017; Chery et al., 2021). The spatial distribution of PCM components is uneven, which suggests its involvement in fine-tuned mechanosensation (Guilak et al., 2006).

Dynamic mechanical loading enhances the gene expression of cartilage-specific transcription factors and ECM components in chondroprogenitor cells and stimulates the chondrogenic differentiation *in vitro* (Takahashi et al., 1998; Elder et al., 2000; Juhasz et al., 2014). Mechanosignals may affect chondrocytes in several ways such as integrin signaling (Loeser, 2014). Mechanical loading of articular cartilage results in activation of ion channels (e.g., stretch or voltage gated channels, big conductance K^+^ (BK), transient receptor potential (TRP), Piezo1/2 channels, etc.) (Zelenski et al., 2015; Lee et al., 2017; Zhang et al., 2021). Primary cilia harbor plasma membrane and signaling proteins, and deformation of these cell surface projections plays a role in chondrocyte mechanotransduction (Williantarra et al., 2022). Nuclear deformation and remodeling of the actin cytoskeleton can also be caused by physical stimuli (Erickson et al., 2003; Swift and Discher, 2014).

**FIGURE 1:**
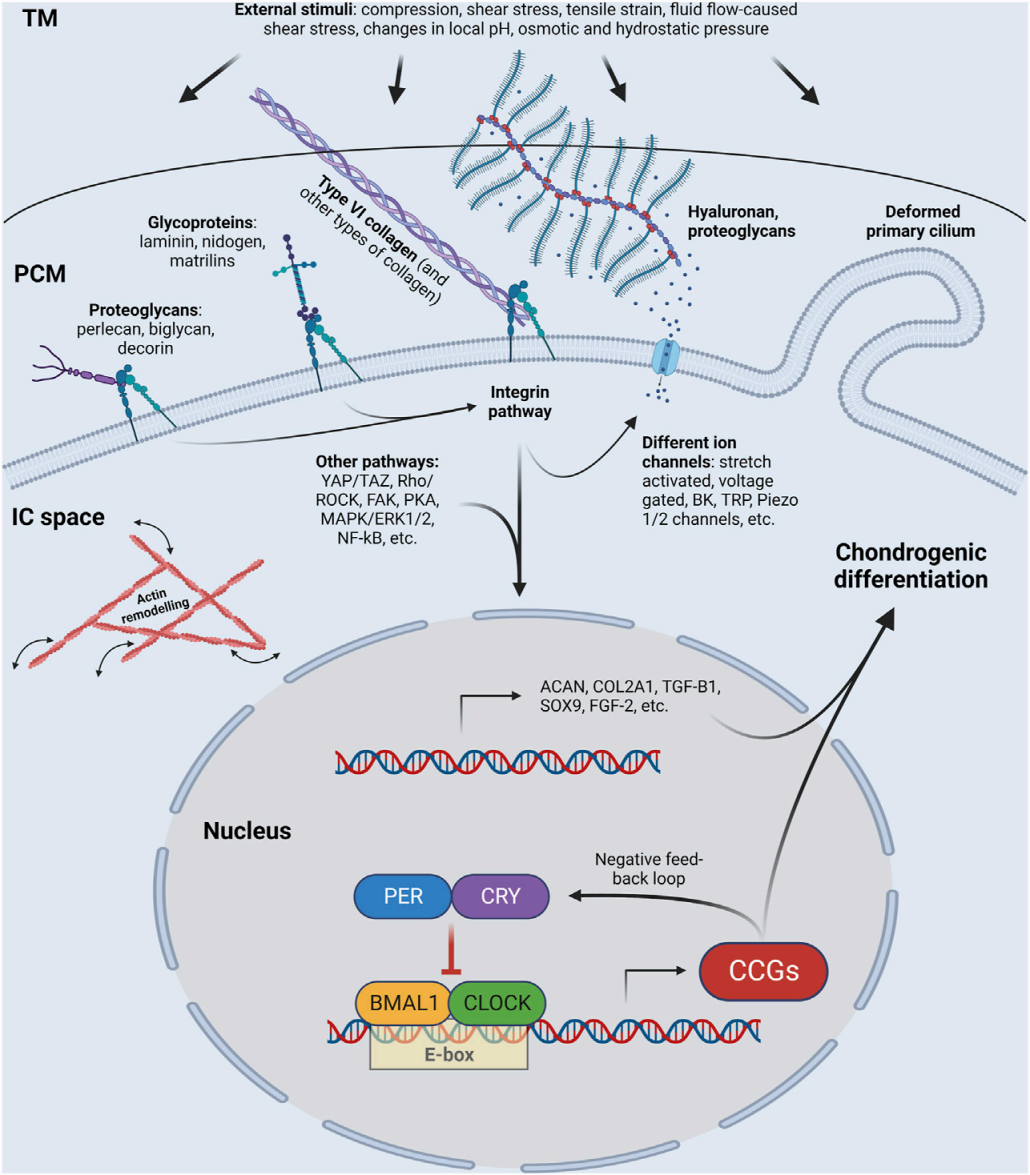
Schematic illustration of a chondrocyte, showing the molecules that sense and transmit external stimuli (such as compression, shear stress, tensile strain, fluid flow-caused shear stress, charges in local pH, osmotic and hydrostatic pressure) from the extracellular matrix via the pericellular matrix into the cytosol, the cytoskeleton, and the nucleus, at least partially via the circadian clock. Some of the core circadian clock genes are shown in the nucleus. Please note that the list of proteoglycans shown in the figure is not exhaustive; other proteoglycans such as fibromodulin and lumican are also important. TM, territorial matrix; PCM, pericellular matrix; IC space, intracellular space; CCGs; clock-controlled genes; BK, big conductance Ca^2+^ activated K^+^ channel; TRP, transient receptor potential channel. See other abbreviations in text. Created with BioRender.com.

Biomechanical signals subsequently activate downstream signaling (Figure 1) (Juhasz et al., 2014; Volz et al., 2022). The increased expression of glycosaminoglycans, type II collagen, SOX9, phosphorylated SOX9 (Juhasz et al., 2014) and growth factors such as transforming growth factor β1 (TGF-β1) and fibroblast growth factor-2 (FGF-2) promote chondrogenic differentiation (Ouyang et al., 2019).

The anatomical characteristics of synovial joints, the types and extent of load, as well as the metabolic status of the body determine articular cartilage fate. Inappropriate mechanical load can alter the reciprocal interaction between chondrocytes and the ECM/PCM (Gilbert et al., 2021), which in turn can contribute to matrix degeneration and mechanically induced inflammation (Lambert et al., 2020); and the PCM may be the first to signal the onset of OA (Chery et al., 2020).

## 4 Cartilage circadian clock

Intrinsically driven circadian rhythms have evolved in response to the external 24-h day-night cycle and result from a network of tissue clocks and rhythms comprised by cellular oscillators (Dibner et al., 2010). These cellular oscillators generating circadian rhythms are expressed in almost every nucleated cell in the body, including the central circadian clock in the suprachiasmatic nucleus (SCN) of the hypothalamus and peripheral clocks such as liver, adipose and cartilage tissues. At the heart of the cellular molecular clock is a genetically conserved autoregulatory feedback loop consisting of transcription factors CLOCK/NPAS2 and BMAL1, driving expression of a plethora of genes, including *Per* and *Cry*, whose protein product interact with the CLOCK–BMAL1 complex and thereby repress their own expression (Takahashi, 2017). The CLOCK–BMAL1 complex also drives output from this molecular oscillation by promoting circadian expression of ∼5–20% of the transcriptome in a tissue-specific context (Zhang et al., 2014).

One of the first mentions of day-night variation in cartilage physiology is a report that the mitotic index in rat cartilage peaks in the morning (Simmons, 1964). Circadian biology in chondrocytes has recently been reviewed (Rogers and Meng, 2023). It has been firmly established that both human and mouse cartilage tissue and chondrocytes express circadian clocks (Gossan et al., 2013; Dudek et al., 2016; Akagi et al., 2017), and that the expression of these circadian clocks develops between days 11–21 of chondrocyte differentiation from stem cells (Naven et al., 2022). Cellular clocks drive circadian rhythms in just under 4% of the mouse cartilage transcriptome (Gossan et al., 2013), which include genes involved in remodeling of the ECM and metabolic homeostasis (Yang and Meng, 2016). The core clock and cartilage marker genes shows a rhythmic expression pattern in mature chondrocytes derived from healthy knee articular cartilage and rib growth plate (Hinoi et al., 2006; Takarada et al., 2012), which indicates that they possess a well-functioning circadian clockwork *in vivo*. Expressing synchronized circadian rhythms in physiology was recently shown to benefit early chondrogenesis (Alagha et al., 2021), which is in line with the general notion that temporal organization benefits physiology.

The benefit of circadian rhythms in cartilage also becomes clear from studies in which the essential clock gene *Bmal1* is ablated. Chondrocyte-specific *Bmal1* ablation in mice associated with lesions in knee cartilage and loss of chondrocytes and ECM, which became more pronounced over time (Dudek et al., 2016). Indeed, in surgical models of OA in mice, cartilage-specific absence of the circadian clock through *Bmal1* knockout leads to more rapid cartilage degeneration than in wildtype mice (Qian et al., 2023). This is, at least in part, associated with a suppression of TGF-β signaling (Dudek et al., 2016; Akagi et al., 2017). Conversely, there are reports that expression of the circadian clocks is perturbed in human cartilage tissue of OA patients (Akagi et al., 2017; Soul et al., 2018), although the timing of the sample collection is unclear as they were acquired from patients undergoing knee surgery, which may affect interpretation.

## 5 Mechanical signals as *Zeitgebers* for the circadian clock

Circadian clocks are influenced and synchronized by internal and external factors (*Zeitgebers*). Dark and light cycles are the most important exogenous *Zeitgeber*, but other external stimuli may also have a significant impact on the clockwork (Gossan et al., 2015). Cells in the peripheral tissues express their own circadian regulation which is synchronized by non-photic cues such as glucocorticoid signaling (Balsalobre et al., 2000; Astiz et al., 2019). For cartilage, mechanical loading is essential for proper differentiation and homeostasis (Fahy et al., 2018). The biomechanical environment of chondrocytes can be influenced through the application of mechanical stimulation, which is required for embryonic cartilage formation and maintaining the healthy biological characteristics of mature cartilage (Sanchez-Adams et al., 2014; Fahy et al., 2018; Volz et al., 2022). Mechanical stimulation in itself promotes the chondrogenic differentiation pathway of MSCs (Fahy et al., 2018). While mechanical stimulation is a key external factor for physiological cartilage metabolism, the molecular details of mechanotransduction pathways are not fully understood.

Given that cartilage is avascular and aneural, the master clock in the hypothalamus is unlikely to be an important synchronizer for the cell-autonomous circadian clocks in chondrocytes. After being cultured *in vitro*, the asynchronous expression of clock genes has been observed during *in vitro* chondrogenesis (Alagha et al., 2021). However, after applying serum shock, clock and chondrogenic marker genes showed a synchronized mRNA expression pattern in differentiating chondrocytes (Alagha et al., 2021). This indicates that the peripheral clockwork can be entrained in chondroprogenitor cells by specific stimuli, acting as local timing cues for the cells.

The molecular clock in chondrocytes is mainly influenced by internal factors such as hormones, growth factors or thermal cues, and also by various external factors (Kamagata et al., 2017; Rogers et al., 2017). Mechanical stimulation can function as a *Zeitgeber* for resetting and entraining the circadian clock in cartilage-specific cells (Kanbe et al., 2006; Yang et al., 2017). Chondrocytes are sensitive to mechanobiological stimuli through mechanoreceptors in their plasma membrane (Lee et al., 2017; Zhao et al., 2020). Uniaxial dynamic compressive force enhances the chondrogenic differentiation of primary chondroprogenitor cells (Vago et al., 2022). The core molecular components of the circadian clockwork, as well as chondrogenic markers showed a synchronized expression pattern after mechanical stimulation, both at mRNA and protein levels, which was otherwise not detectable (Vago et al., 2022). Therefore, dynamic mechanical stimulation served as a *Zeitgeber* for chondroprogenitor clock entrainment, and chondrogenesis was stimulated through the synchronizing ability of the loading regime. When primary articular chondrocytes were exposed to cyclic biaxial tensile stretch, BMAL1 exhibited a sinusoidal expression pattern at the protein level, and the oscillation parameters of BMAL1 followed a daily rhythm which was mimicked by mechanical stimulation (Heywood et al., 2022). Similarly, the molecular clockwork in human dental pulp-derived MSCs could be entrained following rhythmic uniaxial mechanical stretch (Rogers et al., 2017).

The above data confirms that in addition to mature cells, circadian rhythmicity may also be influenced by mechanical cues in undifferentiated MSCs.

## 6 Conclusion

Mechanobiology and chronobiological signaling pathways are closely interconnected during cartilage formation and maintenance. Appropriate mechanical stimuli can serve as external timing cues and entrain the circadian clock in developing (Vago et al., 2022) and mature isolated chondrocytes (Heywood et al., 2022).

However, an important link is still elusive between the biomechanical environment and the chondrocyte clock. While *CLOCK* has been functionally associated with mechanical stress (Kanbe et al., 2006), the mechanotransducers by which the mechanical environment affects the circadian clock have not been fully mapped.

Actin dynamics have been linked to circadian regulation (Hoyle et al., 2017). The circadian clock is influenced by the stiffness of the extracellular environment via vinculin and the Rho/ROCK pathway (Yang et al., 2017). Blocking the Rho-kinase pathway is beneficial for the chondrocyte phenotype and ECM production (Piltti et al., 2017). The CREB/CRE pathway has also been suggested to couple timing cues following mechanical stimuli to the resetting of the circadian clockwork (Heywood et al., 2022). A recent study has confirmed the role of YAP/TAZ in influencing the circadian clockwork by disrupting REV-ERBα oscillations (Abenza et al., 2022).

Ca^2+^ signaling pathways mediated by mechanosensitive ion channels that influence the chondrocyte phenotype may also act as key upstream regulators of the clock genes (Mobasheri et al., 2019; Cavieres-Lepe and Ewer, 2021). Mechanical stimuli can affect mechanosensitive ion channels, and the resulting ionic fluxes then modulate chondrocyte metabolism (Zhang et al., 2021). Ca^2+^ influx via N-methyl D-aspartate (NMDA) receptors has recently been shown to regulate the circadian clock components PER2 and BMAL1 in chondrocytes through activation of the CREB and NF-κB signaling pathways (Kalev-Zylinska et al., 2018; Alhilali et al., 2021).

## 7 Perspectives

The current insights into the chronobiology of cartilage biology inspire at least two important future directions. One is aimed at the chronobiology of clinical treatments and tissue engineered cartilage grafts, and another aimed at understanding the mechanistic links between the molecular physiology of cartilage and circadian clocks.

The majority of currently marketed medicinal products may benefit from chronotherapy, a timed administration based on the circadian rhythmicity of the drug target (Lee et al., 2021). It is known that many drug targets exhibit circadian rhythmicity (Zhang et al., 2014), and the targets for treatment of OA could have similar patterns. It would also be advantageous to consider the time of day/circadian phase of stem cell-based therapies. An improved understanding of the interactions between chronobiology and the pathomechanisms of OA pain would enhance targeted drug discovery programs, resulting in the development of better therapeutic strategies.

However, circadian rhythmicity is currently not being exploited for cartilage tissue engineering approaches, despite the emerging role of the biological clock in developing and mature chondrocytes in health and disease. Understanding the circadian physiological landscape in cartilage cultures, and the contrast between *in vitro* clock synchronization methodologies (such as dexamethasone or serum shock) and mechanical stimulated cultures will give key insights into the molecular links between cartilage molecular physiology and the circadian timing system. Unveiling the details of how mechanoreception sits at the intersection of cartilage formation and molecular circadian oscillators will give us putative targets for the optimal time of day of treatment. Combining this with the effects of biomechanics on chondrocyte metabolism (i.e., metabolomics) would help in the successful development and clinical translation of tissue-engineered cartilage grafts to restore joint function, delaying the need for prosthetic interventions.
